# Supplementary figures and images for: The deciduous genital spines of the moth Peridea anceps (Goeze, 1781): Potential socially transferred materials
Source: PLoS One. 2025 Sep 3;20(9):e0329104. doi: 10.1371/journal.pone.0329104 (PMC12407467; doi:10.1371/journal.pone.0329104)

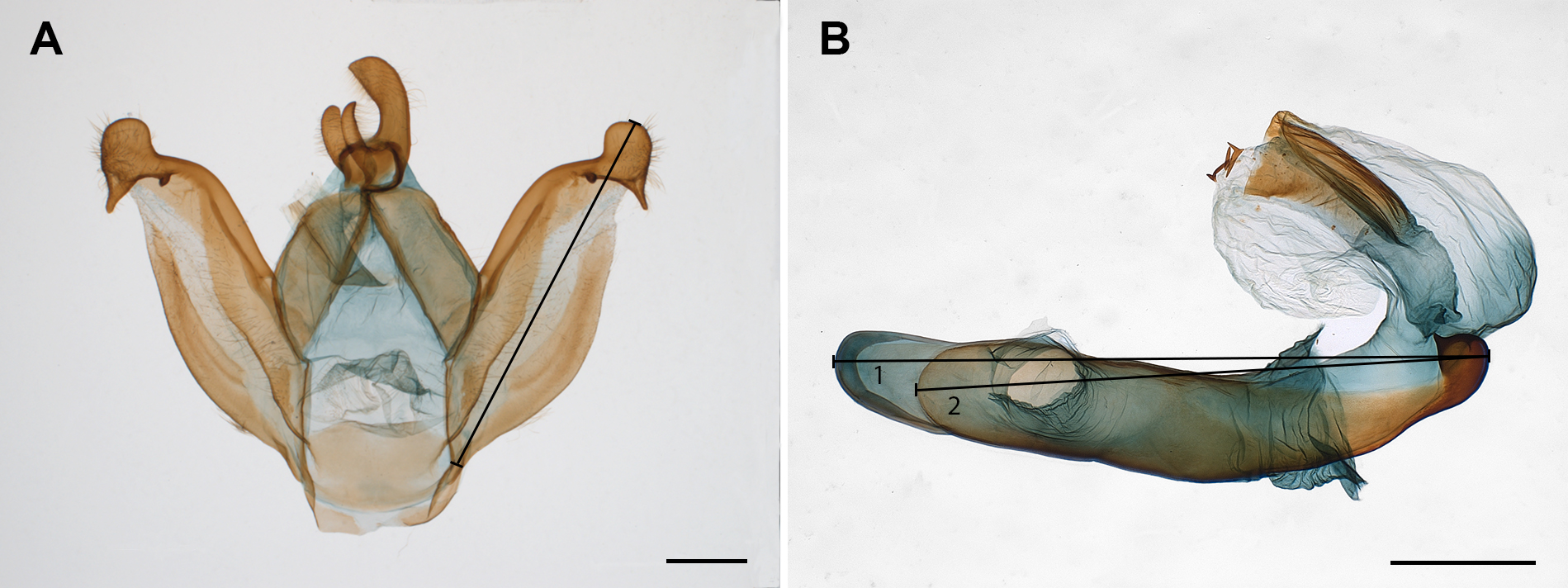

Supplement: S1 Fig — (JPG) [file pone.0329104.s001.jpg]

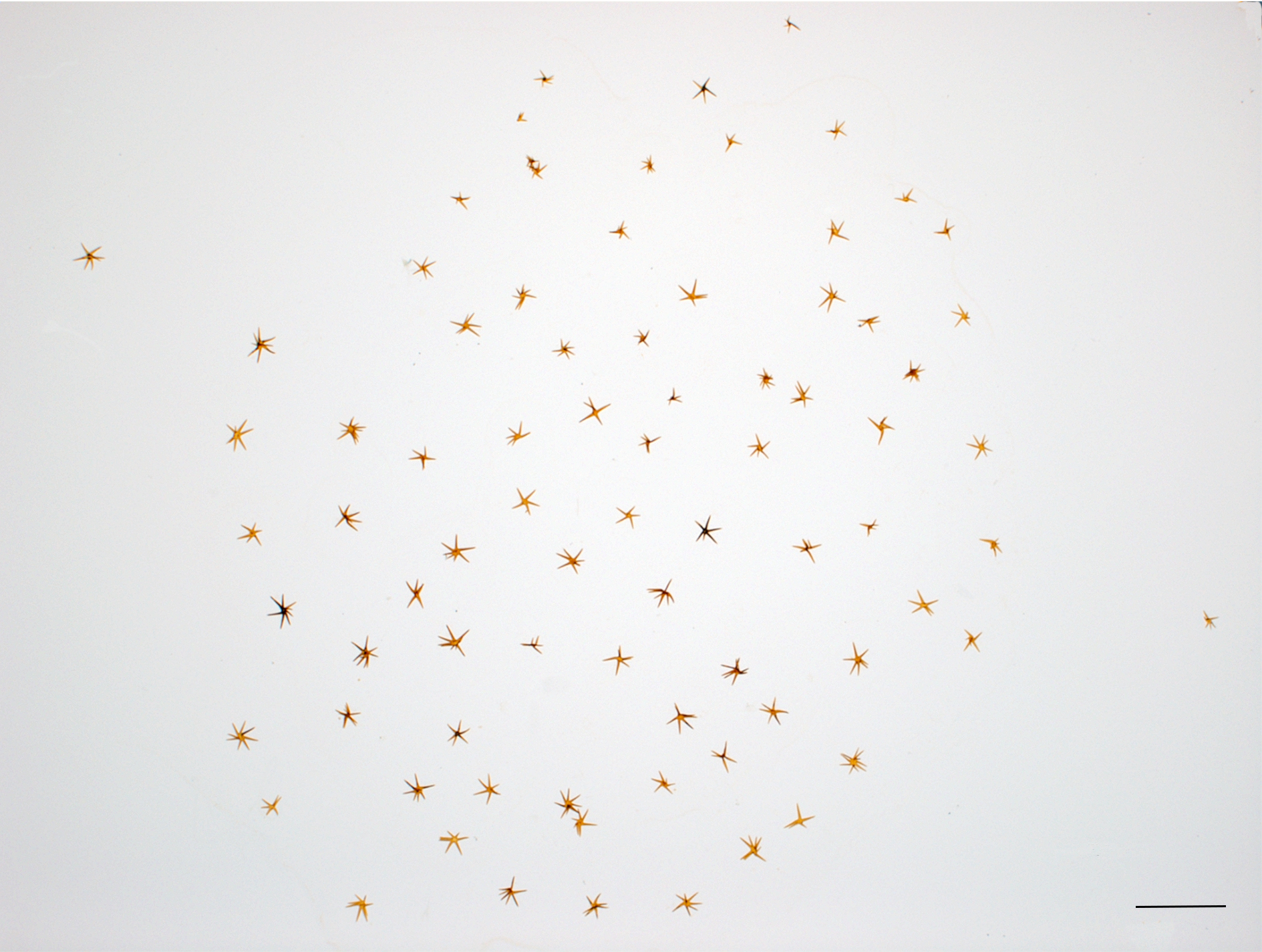

Supplement: S2 Fig — (JPG) [file pone.0329104.s002.jpg]
