# Supplementary material for: The deciduous genital spines of the moth Peridea anceps (Goeze, 1781): Potential socially transferred materials
Source: PLoS One. 2025 Sep 3;20(9):e0329104. doi: 10.1371/journal.pone.0329104 (PMC12407467; doi:10.1371/journal.pone.0329104)
Supplement: Attachment — Submitted filename: PLOS ONE Registered Report Protocol Reviewer Form.pdf [file pone.0329104.s009.pdf]

**Reviewer Recommendation and Comments for Manuscript Number**

Original Submission

Recommendation: No Recommendation

Cancel | Save & Submit Later | Upload Reviewer Attachments | Proof & Print | Proceed

**Reviewer Instructions**

To complete your review, answer the questions in the review form below. (A red asterisk (*) indicates that a response is required.) Enter the main body of your review under question 5, along with any concerns about potential competing interests on the part of the authors, data availability, or research ethics. Declare your own potential competing interests under the section titled Confidential to Editor. This information is visible only to the editor and the journal office.

**Confidentiality**

The review process is strictly confidential. If you become aware of any issue that could potentially compromise the integrity of the process, contact the journal before consulting with colleagues.

**Published peer review history**

If this manuscript is accepted for publication, the authors will have the option to publish the peer review history alongside the final article. The peer review history package includes the complete editorial decision letter, with the reviews, for each version of the manuscript, as well as the authors' responses to reviewers.

**Anonymity**

Your identity will not be revealed to the authors unless you sign your review, or upload an attachment that has your name in the file name or contents. If you choose to sign your review, your name will appear in the editorial decision letter where the authors and other [illegible]

**Review Questions**

***Comments to the Author**

Insert Special Character

1. Does the manuscript provide a valid rationale for the proposed study, with clearly identified and justified research questions?

The research question outlined is expected to address a valid academic problem or topic and contribute to the base of knowledge in the field.

Please select a response

---

*2. Is the protocol technically sound and planned in a manner that will lead to a meaningful outcome and allow testing the stated hypotheses?

The manuscript should describe the methods in sufficient detail to prevent undisclosed flexibility in the experimental procedure or analysis pipeline, including sufficient outcome-neutral conditions (e.g. necessary controls, absence of floor or ceiling effects) to test the proposed hypotheses and a statistical power analysis where applicable. As there may be aspects of the methodology and analysis which can only be refined once the work is undertaken, authors should outline potential assumptions and explicitly describe what aspects of the proposed analyses, if any, are exploratory.

Please select a response

---

*3. Is the methodology feasible and described in sufficient detail to allow the work to be replicable?

---

*4. Have the authors described where all data underlying the findings will be made available when the study is complete?

The PLOS Data policy requires authors to make all data underlying the findings described in their manuscript fully available without restriction, with rare exception, at the time of publication. The data should be provided as part of the manuscript or its supporting information, or deposited to a public repository. For example, in addition to summary statistics, the data points behind means, medians and variance measures should be available. If there are restrictions on publicly sharing data—e.g. participant privacy or use of data from a third party—those must be specified.

---

*5. Is the manuscript presented in an intelligible fashion and written in standard English?

*PLOS ONE* does not copyedit accepted manuscripts, so the language in submitted articles must be clear, correct, and unambiguous. Any typographical or grammatical errors should be corrected at revision, so please note any specific errors here.

---

*6. Review Comments to the Author

Please use the space provided to explain your answers to the questions above and, if applicable, provide comments about issues authors must address before this protocol can be accepted for publication. You may also include additional comments for the author, including concerns about research or publication ethics.

You may also provide optional suggestions and comments to authors that they might find helpful in planning their study.

(Please upload your review as an attachment if it exceeds 20,000 characters) (Limit 100 to 20000 Characters)

Character Count: 0

---

*7. PLOS authors have the option to publish the peer review history of their article (what does this mean?). If published, this will include your full peer review and any attached files.

If you choose "no", your identity will remain anonymous but your review may still be made public.

**Do you want your identity to be public for this peer review?** For information about this choice, including consent withdrawal, please see our Privacy Policy.

Yes

**Response required**
Your full name*, as it should appear in the published peer review:

*Please do not sign this review on behalf of another person. (Limit 2 to 20000 Characters)

Character Count: 0

---

***Confidential to Editor**

1. Do you have any potential or perceived competing interests that may influence your review? Please review our Competing Interests policy and declare any potential interests that you feel the Editor should be aware of when considering your review. If you have no competing interests, please write "I have no competing interests." (Limit 20000 Characters)

Character Count: 0

2. Did you receive any assistance in preparing this review (e.g. from a post-doc or graduate student)? If yes, please provide his/her name and title here. If you have chosen to reveal your identity to the authors in question 6 above, please include the name of the person who assisted you in the response to question 6 as well. (optional) (Limit 200 Characters)

Character Count: 0

3. If accepted, do you think this submission should be highlighted on the PLOS ONE website? PLOS ONE does not evaluate manuscripts based on perceived significance or readership. We aim to provide tools for readers to filter and evaluate our publications. (optional)

Please select a response

**ORCID Reviewer Credit**

**Note:** This reviewer's default response is 'No' so this Review will not be sent to ORCID; you cannot change this as you are proxied in for this person. The Reviewer may change this after you submit the review, by going to their Completed Reviewer Assignments folder and using the 'ORCID Deposit Authorization' action link. They must log in to do so - you cannot do that by proxy.

Cancel | Save & Submit Later | Upload Reviewer Attachments | Proof & Print | Proceed
